# Work and Economy in War-Time: How to Secure Both by Co-Operation

**Published:** 1915-12-18

**Authors:** 


### WORK AND ECONOMY IN WAR-TIME.

### How to Secure both by Co-operation.

This, the present Special Number, has been prepared and organised with a view to help every hospital worker and every hospital supporter to put their shoulders at the wheel to advance the business. Now what is the business of voluntary hospitals at this stage of the war, with the disarrangement of the civil organisation of hospitals by the inrush of wounded sailors and soldiers? Clearly it is to unite all hands, to direct attention to everything calculated to help forward the prosperity of the voluntary hospitals, the efficiency of their work, the speedy recovery of the patients, and the comforting and strengthening of all hospital workers and supporters everywhere. A reference to the first article in the present issue will encourage many, we hope, to make it their business to lend a hand to some hospital, and, having set their shoulders at the wheel, to advance the business. To-day's issue of The Hospital contains many indications of how to set to work and what to attempt.

Our first suggestion is that at each hospital some, or all, of the following new methods should be pursued. The urgent need for economy, and the existence of the great war, should be taken advantage of to overhaul, as at St. Thomas's Hospital, the departmental methods existing, with a view to improvement wherever possible. Thus there is a distinct call for better knowledge and definite technical teaching for all who have to do with the upkeep, selection, and maintenance of the domestic branches of hospital administration. We have endeavoured to indicate one way to secure this end in the preliminary article published on page 255, which treats of textiles in the fullest meaning of that word and their supply to our hospitals. Anyone who studies the article and considers the ground to be covered in the series given on page 256, to commence from the first number of the New Year, will be able in some measure to realise the importance of the issues here raised and to be raised.

Again, there is interest and an indication of new ground in the article on "A War Hospital Supplies Depôt" well worthy of attention. Another important field for the practice of economy is that of galenicals. It is not so widely known or appreciated as it should be, but in the case of some hospitals of importance, as, for instance, the Royal Victoria Infirmary, Newcastle-on-Tyne, by undertaking the manufacture of many galenicals and tablets for use in the treatment of the patients by the honorary medical staff great savings have resulted. On an expenditure of some £1,600, representing the cost of purchasing these various articles at the prices given in the wholesale lists, after deducting 20 per cent., a net saving for the year of upwards of £770 has been effected. We propose in the New Year to point the way for other hospitals, which possess an able staff and ambitions to do special and good service during war-time, to reconsider the basis on which they at present purchase their galenicals, with a view to effect similar savings to those resulting from a change of system at Newcastle and elsewhere.

In matters of equipment we hope, with the co-operation of those who have articles and methods which possess special merits or secure distinct advantages in the working of departments or wards, and that tend to produce economy in labour, in time, and other directions, to give a full description, with illustrations, of their appliances and their fruit, to enable everyone to judge each upon its merits. The need for information and technical knowledge in this field of work may be appraised by those who refer to "A Layman's Account of a Ward's Equipment," which appears on page 266.

We desire specially to direct attention to the articles and illustrations which demonstrate the practical interest and value of the creation of Mr. William Pite, the distinguished architect of the new King's College Hospital buildings at Denmark Hill. Here we have an entirely new modern hospital, an old foundation removed from the centre of London, placed upon an airy and, as town sites go, a fairly large site, where everything has been done to secure the best possible, and to admit nothing, however novel, which has not had the full test of reasonable trial and exhibited distinct advantages. We hope that in due course the new King's College Hospital buildings, their equipment and administration, when they revert entirely to the purposes of a civil hospital, may become the Mecca of the hospital world, at any rate until the lessons it can teach are fully mastered and understood.

Finance is an all-important note of vital interest to the well-being of every voluntary hospital. We would especially direct the attention to the article entitled "Hospital Needs and Appeals," in the Editor's Letter-Box, and the striking figures there given; to the sermon by Bishop Frodsham, Canon of Gloucester, dealing with the Hospital Sunday Fund and its teachings, which enforces the importance to voluntary hospitals and benevolent institutions of outside effort to arouse the interest and support of the general public.

Last, but by no means least, we ask the authorities of every hospital, and hospital officers generally, to study with attention the article entitled "Every Hospital's Own Gazette." The great success the *Gazette* idea has secured already in connection with military hospitals, the outpouring of talent by able literary men and artists, and the undoubted and increasing literary success secured for such ventures, suggests a new way to popularise the voluntary hospital, to make its value more widely appreciated, to secure for it an ever-increasing revenue, and to surround it with an always growing phalanx of keen workers in support of its every requirement and need.

